# Longitudinal monitoring and prediction of long-term outcome of scar stiffness on pediatric patients

**DOI:** 10.1093/burnst/tkab028

**Published:** 2021-09-30

**Authors:** Bettina Müller, Edoardo Mazza, Clemens Schiestl, Julia Elrod

**Affiliations:** Institute for Mechanical Systems, Department of Mechanical and Process Engineering, ETH Zurich, Leonhardstrasse 21, 8092 Zurich, Switzerland; Institute for Mechanical Systems, Department of Mechanical and Process Engineering, ETH Zurich, Leonhardstrasse 21, 8092 Zurich, Switzerland; Empa, Swiss Federal Laboratories for Materials Science and Technology, Ueberlandstrasse 129, 8600 Dübendorf, Switzerland; Burn Center, Division of Plastic and Reconstructive Surgery, University Children's Hospital Zurich, Steinwiesstrasse 75, 8032 Zürich, Switzerland; Children's Research Center (CRC), University Children's Hospital Zurich, Steinwiesstrasse 75, 8032 Zürich, Switzerland; Burn Center, Division of Plastic and Reconstructive Surgery, University Children's Hospital Zurich, Steinwiesstrasse 75, 8032 Zürich, Switzerland; Children's Research Center (CRC), University Children's Hospital Zurich, Steinwiesstrasse 75, 8032 Zürich, Switzerland

**Keywords:** Pediatric burns, Scar maturation, POSAS, Cutometer, Nimble

## Abstract

**Background:**

Hypertrophic scarring after burn injury is one of the greatest unmet challenges in patients with burn injuries. A better understanding of the characteristics of scar maturation and early prediction of the long-term outcome of scarring are prerequisites for improving targeted therapies and pivotal for patient counselling.

**Methods:**

Repeated measurements of scar stiffness in 11 pediatric patients were performed over the course of 1 year using 2 suction devices, the Cutometer and the Nimble. In addition, the observer pliability score of the Patient and Observer Scar Assessment Scale was applied. This longitudinal study allowed quantification of the ability of each of the measured parameters to reflect scar maturation, as indicated by change in skin pliability/stiffness, over time (using linear regression); the ability to distinguish individual patients (intraclass correlation coefficient (ICC)); the correlation of the devices (Spearman correlation coefficient); and the ability to predict long-term scar maturation based on early scar assessment (using receiver operating characteristic).

**Results:**

All the tools used showed significant longitudinal decrease of scar stiffness from 3 months until 12 months after the injury. The Nimble (ICC_patient_^Nimble^ = 0.99) and the Cutometer (ICC_patient_^Cuto^ = 0.97) demonstrated an excellent ability to distinguish between individual patients. The Nimble seemed to be able to predict the 12-month pliability of scars based on early (3-month) measurements (area under the curve (AUC)_12m_^POSAS^ = 0.67; AUC_12m_^C^ = 0.46; AUC_12m_^N^ = 0.79).

**Conclusions:**

The results of this preliminary study suggest that all 3 tools provide suitable means to quantify alterations in scar stiffness over time. Initial evidence suggests the Nimble is most favorable for predicting changes in stiffness associated with long-term scar maturation. Further studies with a larger sample size are required to validate tissue suction as a clinical tool for analysis of changes of scar stiffness over time.

Highlights

- This study systematically investigates longitudinal maturation of pediatric burns by means of suction measurements (Cutometer, Nimble) and by means of the POSAS.
- This study suggests objective, non-invasive measurement tools to be able to predict long-term scar outcome based on an early scar assessment.
- Results indicate the potential of the Nimble to objectively describe scar maturation in order to answer scientific questions regarding scar treatment.

## Background

A significant percentage of burns in pediatric patients result in hypertrophic scarring that may entail substantial functional disability and esthetic impairment, depending on the depth, extent and anatomical location of the burn [1, 2]. In many cases, scars lead to a reduction in quality of life (QOL) and the need for numerous corrective surgeries, which additionally lead to a substantial economic burden [3–5]. In fact, as the probability of surviving acute burns has significantly improved over the past decades, hypertrophic scarring is now one of the greatest unmet clinical challenges in patients with burn injuries [1]. Besides large thermal injuries, even small ones in delicate regions, such as contact burns on the palm of the hand or facial injuries, can lead to debilitating functional deficits, poor esthetic outcomes and a negative psychological impact, necessitating intensive follow-up care or leading to a reduced QOL [6–8].

Hypertrophic scars and keloids develop within the first few weeks to months after an injury, after which, in many cases, a spontaneous partial involution is observed [9]. Scar maturation has not been sufficiently investigated and is only partially understood [10, 11]. The extent of (hypertrophic) scarring seems to depend on many factors, such as the nature of the underlying injury and the anatomic region, but also demographic factors, such as skin pigmentation and genetic disposition. Furthermore, wound infection and prolonged healing negatively affect scar outcome [12–14].

A better understanding of the characteristics of scar maturation is important for several reasons. First, an in-depth understanding would support the prediction of scar outcomes after burns. This could improve decision making and patient counselling with regard to early scar interventions versus conservative management after severe burns. Second, a thorough comprehension would facilitate the distinction of spontaneous scar maturation over time from therapy-induced improvement. This would enable determination of the true efficacy of distinct scar therapies, whereas, using current assessment methods, evidence in many areas of burn treatment remains poor [15]. Third, scar functional characterization would improve our knowledge of the biology behind this fibrotic process, which is a prerequisite for the development of targeted therapies.

Research on scar maturation in the context of thermal injuries is further complicated by the lack of a homogenous use of the term ‘maturation’ itself, with its past use in the context of studies performing long-term assessments of scars being quite heterogeneous [16–20]. However, it seems clear that the processes of maturation within the first few months to years after a burn are complex and involve both histological characteristics [16] and macroscopically quantifiable features, such as those included in the Patient and Observer Scar Assessment Scale (POSAS).

In the past, great effort was directed towards the development of objective measurement tools that can be used to describe biomechanical features of scars *in vivo*. The suction method is one of the most commonly used measurement techniques [21]. The principle of this is based on drawing the skin into a geometrically defined circular opening through generation of a vacuum. The DermaLab (Cortex Technology, Denmark) is a displacement-controlled suction device that has shown good reliability and reproducibility in measurements of healthy skin [22, 23], aging skin [24] and scar tissue [25, 26]. The same studies report that a limitation of the DermaLab is the high deformation levels it induces, which impede measurement of the very stiff tissues that sometimes occur in extremely hypertrophic scars. However, if required, a special suction cup with reduced elevation levels can be used to measure pronounced skin fibrosis [27]. A second commercial suction device, the Cutometer (Courage & Khazaka Electronics GmbH, Germany), is widely used in the cosmetic industry [28, 29] for the assessment of hypertrophic scars [30, 31] and quantification of skin fibrosis [32, 33]. This device operates via a load-controlled mechanism and uses an optical measurement system to track the elevation of the tissue. The Cutometer does have some limitations regarding the interaction between the observer and the probe during the measurement [34, 35]. To overcome these limitations, we developed a novel, ultra-light, displacement-controlled suction device (Nimble). Recently, its suitability for measuring healthy skin [36] in pediatric patients suffering from hypertrophic scars after burn wounds [37] and its ability to distinguish between the skin stiffness of patients with systemic sclerosis and the skin stiffness of healthy controls was demonstrated [38].

The aim of this prospective investigation in pediatric burn patients was to compare the performance of the novel suction device, the Nimble, with established and validated scar characterization methods and to quantify scar maturation in terms of alterations of biomechanical characteristics of skin over time. For this purpose, pediatric burn scars were monitored repeatedly using the investigational device, the Nimble, the widely used suction device, the Cutometer, and the POSAS, a validated but subjective scar assessment method, at defined points of time for 1 year.

**Figure 1.**
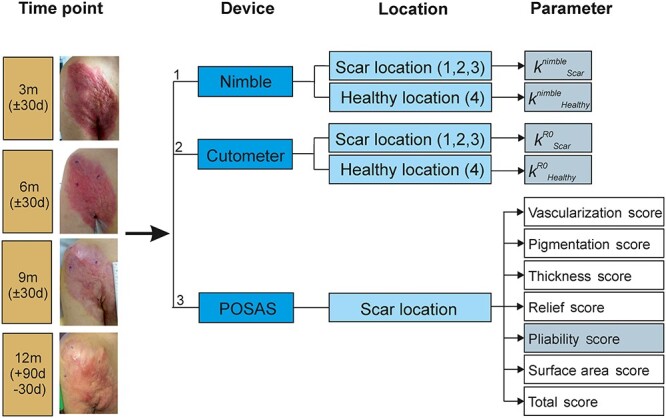
Measurement scheme. At every visit (3, 6, 9 and 12 months after the injury or transplantation) the stiffness of the scar (see Figure 2b, locations 1, 2 and 3) and the healthy skin (see Figure 2a, location 4) was measured with the Nimble and the Cutometer. Afterwards, the Patient and Observer Scar Assessment Scale (POSAS) was completed by the same observer and the patient or caregiver. *m* months, *d* days, *k* tissue stiffness, *R0* maximum elevation parameter

## Methods

This study was approved by the Swiss Agency for Therapeutic Products (Swissmedic, 2017-MD_0039) and by the local ethics committee (Kantonale Ethikkommission Zurich, KEK-ZH-Nr. 2017-02015).

### Study cohort

For this study, 11 pediatric patients presenting to the outpatient department of the University Children’s Hospital of Zurich were recruited. The admission of the patients was in accordance with the inclusion criteria (recent thermal injury with or without skin transplant; age ≥1 year and ≤18 years; signed informed consent) and exclusion criteria (concomitant medical conditions that could interfere with wound healing; inability to follow the procedures, e.g. due to language problems; persisting unhealed wounds at or adjacent to the measuring sites; enrollment of family members of the study staff or employees of the hospital).

### Scar assessment procedure

Study visits took place at 3 months (±30 days), 6 months (±30 days), 9 months (±30 days) and 12 months (+90 days or −30 days) after injury or after the time of transplantation, whenever applicable (Figure 1).

**Figure 2.**
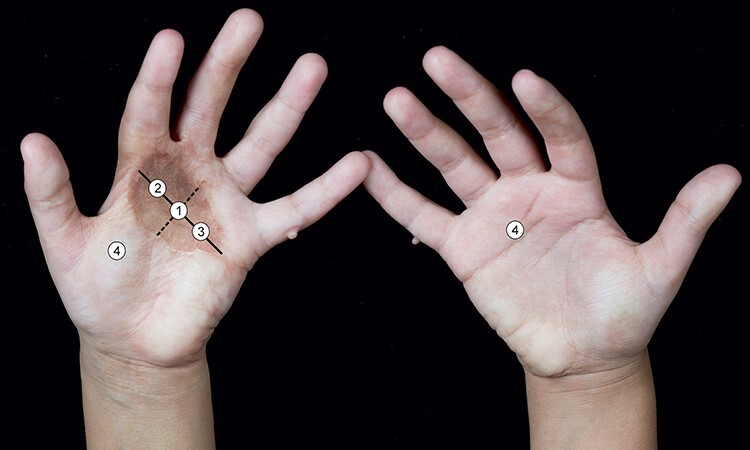
Locations of the points measured with the Nimble and the Cutometer. Location 1 is the intercept of the longitudinal and the transverse axis and points 2 and 3 are located at half distance. An anatomically comparable site on the healthy skin (location 4) was chosen

At each visit, an observer first performed suction measurements with the investigational device, the Nimble, then with the commercial solution, the Cutometer [39], and finally applied the validated scar assessment tool, the POSAS. The stiffness measurements using the suction devices were performed at 3 predefined locations of the scar and 1 location on healthy skin in triplicate. To ensure independence of repetitive measurements on the same skin location a waiting time of at least 45 seconds, based on previous data concerning tissue recovery after suction experiments [40], was guaranteed. In order to enable the comparability of the stiffness values of the 2 devices, guarantee that measurements would be performed at the same locations on the scar at every visit and avoid selection bias, the allocation of the same 3 measuring points was performed in a standardized manner, adopted from others [41]. The measuring point on the uninjured skin was located on the contralateral side of the scar tissue whenever possible, or alternatively was adjacent to the scar, as shown in Figure 2.

### Measurement tools

**Nimble** The Nimble is a suction device with a lightweight probe (3.5 g) [36]. Its measuring principle is displacement-controlled, meaning that the tissue is drawn into the opening of the probe (diameter = 6 mm) to a predefined elevation, *h* (here, *h* = 0.5 mm). The pressure needed for this to happen (closing pressure, *p_cl_* in mbar, where 1 mbar = 10^5^ Pa) is recorded and the tissue stiffness, *k,* is calculated (Table 1). In our study, the time for 1 measurement was 2–15 seconds, depending on the *p_cl_*. Further details concerning the device, its settings and mode of application are described in our previous publications [35–37].

**Table 1:** Summary of absolute stiffness parameters and relative stiffness parameters for both suction devices the Nimble and the Cutometer

**Outcome parameters**			
Absolute stiffness		Relative stiffness	
Nimble	Cutometer	Nimble	Cutometer
${k}^{Nimble}=\frac{p_{cl}}{0.5 mm}$	${k}^{R0}=\frac{250\ mbar}{R{0}_{corr}}$	${q}^N=\frac{k_{Scar}^{Nimble}}{k_{Healthy}^{Nimble}}$	${q}^C=\frac{k_{Scar}^{R0}}{k_{Healthy}^{R0}}$

**Cutometer** The Cutometer is a widely used tool for the assessment of scar stiffness. It is a pressure-controlled suction device, which means that a predefined negative pressure (*p_max_*) is applied to the tissue, resulting in a certain elevation of the skin (*R0*). This elevation is recorded by means of an optical measurement system and allows for the calculation of the skin stiffness as described in Table 1. In this study, the same settings were applied as previously described by our group [36] (mode 2 (continuous loading and unloading), load = 15 mbar/second, maximum suction pressure *p_max_* = 250 mbar, probe diameter = 6 mm). As a result, in the present study, the time for 1 measurement was always 17 × 2 seconds = 34 seconds. The offset, which is the pre-deformation resulting from the contact force between the Cutometer and the skin, was recorded for each measurement. In previous investigations, measurement variability and uncertainties in measurement outcome have been related to the offset in Cutometer measurements [35]. Therefore, a correction scheme that was demonstrated to improve these issues significantly was introduced in this trial [36]. For the reported Cutometer stiffness, this correction procedure was applied. The scheme accounts for the pre-deformation by adding the value of the offset to the measured tissue elevation. Therefore, the maximal elevation, *R0*, is corrected to ${R0}_{corr}=R0+ Offset$. The weight of the handheld device is 165 g [39]. The time for 1 measurement was around 35 seconds.

For both the Cutometer and the Nimble, the quotient of the stiffness of the respective scar over the stiffness of the healthy skin was used to determine the relative stiffness, resulting in the following notations (Table 1). These relative stiffness measures were later used to analyse the longitudinal changes at 6, 9 and 12 months and compare them to the baseline measurement at 3 months after injury.

**POSAS** The POSAS questionnaire is a reliable and validated tool consisting of 2 numerical 10-point scales that are completed by the patient—with the assistance, if needed, of his or her caregiver (parameters: pain, itching, color, stiffness, thickness, irregularity and overall score) —and the study investigator (parameters: vascularization, pigmentation, thickness, relief, surface area, pliability and total score) [42–44].

### Statistics

Statistical analysis was performed using the Python library scipy.stats (Python Software Foundation, USA). Descriptive statistics are reported in the form of boxplots or as means and standard deviations, as applicable. Intraobserver variability, in terms of the ability to distinguish between individual patients, was determined by means of the intraclass correlation coefficient (ICC) (2,k) (ICC_patient_^Nimble^, ICC_patient_^Cuto^) [45]. Analysis of the longitudinal change of scar properties was performed using a linear regression fit for each patient with a centered predictor (time). The significance of this longitudinal change was then tested with a one-sided paired *t*-test. The slopes of the fitted lines were tested for their difference from a horizontal line (slope n = 0), which indicates no longitudinal change of scar properties. Correlation between suction outcome parameters and the POSAS observer pliability score was evaluated using the Spearman correlation coefficient with the following interpretation: 0 < *r* < 0.35 indicates weak correlation, 0.36 < *r* < 0.67 indicates moderate correlation and 0.68 < *r* < 1.0 indicates high correlation between the outcome measures, according to Taylor [46]. To determine whether the long-term (12-month) outcome of the scars is predictable based on early measurements of stiffness with the suction devices or pliability assessment using the POSAS score, the correlation of the POSAS observer pliability scores between 3 months and 12 months indicated 2 groups of scars: Group1, moderate scar stiffness, and Group2, high scar stiffness. For this data, a receiver operating characteristic (ROC) analysis was performed and the area under the curve (AUC) was calculated for each device, so as to determine the sensitivity of each of the tools to predict scar maturation in terms of alterations in scar stiffness or pliability, respectively. Additionally, the most suitable cut-off value (to determine which scar belonged to which group) was calculated using the Youden’s index, *J*.

## Results

### Demographics and injury-related details

All 11 pediatric patients included in the study were evaluable and no dropouts were recorded. Five of them were female. The mean age at time of injury was 6.6 ± 5.7 years (14.4 months to 15.9 years). Nine patients had a Fitzpatrick skin type 1–3, 2 had type 6 skin. Regarding the trauma mechanism, 3 had sustained a scald injury, 6 had suffered a contact burn and 2 had a flame injury. Mean TBSA was 6.9% ± 6.0% (range, 1–21%). Four scars were located on the extremities, 4 on the hands and feet and 3 on the trunk. Six patients underwent skin grafting and 5 healed conservatively. Concerning scar prophylaxis, all patients were treated according to our standard procedure, which includes moisturizers and silicone products in all patients and pressure garments whenever applicable. Patient demographics are summarized in Table 2.

**Table 2:** Study participants and characteristics of burned areas

**Characteristics**	**Participants (*n* = 11)**
Female gender, n (%)	5 (45.5%)
Fitzpatrick skin type, n (%) Type 1–3 Type 6	9 (81.8%) 2 (18.2%)
Mean age of patient at time of injury	6.6 ± 5.7 years (14.4 months to 15.9 years)
Mean age of scar at each visit Visit 2 Visit 3 Visit 4 Visit 5	87.8 ± 13.6 days 174.3 ± 20.2 days 265.6 ± 18.9 days 377.2 ± 23.0 days
TBSA	6.9 ± 6.0% (1–21%)
Etiology, n Scald Flame Contact (including hot fat)	3 2 6
Scar location, n Extremity Hand or foot Trunk	4 4 3
Primary treatment, n STSG Conservative	6 5
Treatment of scar, n (%) Moisturizers Silicone-containing products Pressure garment	11 (100%) 10 (90.9%) 10 (90.9%)

Data are presented as mean ± SD (range)

### Ability of the tools to reflect scar maturation over time and discriminate between time points and inter-patient distinguishability

**Figure 3.**
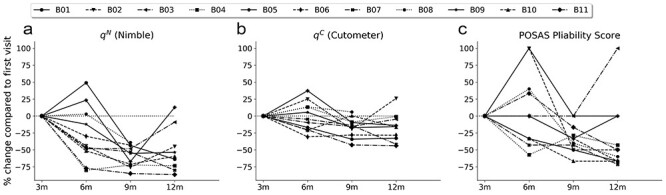
Longitudinal course of relative tissue stiffness measured by the Nimble **(a)** and the Cutometer **(b)** and results of the observer pliability score of the Patient and Observer Scar Assessment Scale (POSAS) **(c)** over the course of 1 year, indicated as percentage changes with respect to the initial visit at 3 months. Suction measurements were conducted by the same observer on 3 defined locations on the scar and 1 location on the healthy skin at each visit. *q* means relative stiffness measure, *B01-B11* indicate patients, *m* months

The results of the longitudinal measurement using the Nimble and the Cutometer and the scar assessment by means of the POSAS observer pliability score performed at 3 months (±30 days), 6 months (±30 days), 9 months (±30 days) and 12 months (+90 days or −30 days) are depicted in Figure 3a, b. c. The raw data of the Nimble, Cutometer and POSAS measurements can be found in Tables S1–S3. For both suction devices, the results are shown as the mean relative tissue stiffness for each patient normalized to the result of the first visit at 3 months. In addition, the POSAS observer pliability results are shown normalized to the first visit as a percentage. All parameters showed qualitative improvements at the 12-month assessment compared to the first visit for most of the patients. Regarding the POSAS patient scale, both the median pliability and the median total score of all patients decreased over time; however, considering patients individually reveals an inconsistent course over time (data not shown). All information on the longitudinal course of the POSAS parameters, including the scores for vascularization, pigmentation, thickness, relief, surface area, pliability and the total score, are summarized in the online supplementary material (Figure S1).

**Figure 4.**
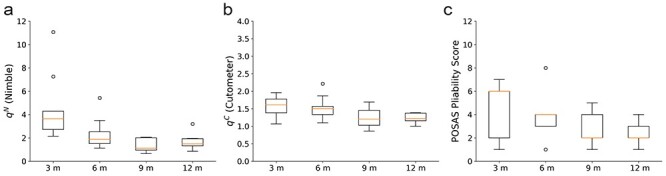
Repeated detection of scar maturation indicated by change in skin pliability/stiffness over the course of 1 year, using the Nimble **(a)**, the Cutometer **(b)** and the pliability score of the Patient and Observer Scar Assessment Scale (POSAS) **(c)**. Suction measurements were conducted at 3 defined locations on the scar and one location on the healthy skin at each visit. For this visualization, the relative stiffness (*q*^N^ and *q*^C^, respectively) was calculated

**Figure 5.**
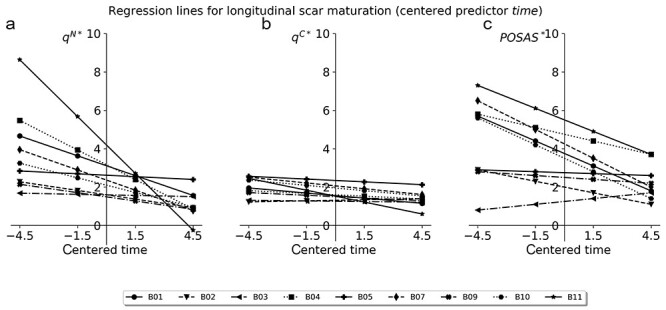
Fitted regression lines for **(a)** measurement outcome of Nimble measurements, **(b)** measurement outcome of Cutometer measurements and **(c)** outcome of the observer pliability score of the Patient and Observer Scar Assessment Scale (POSAS) for 9 out of the 11 evaluated patients. The regression is plotted over the centered time predictor and the slopes indicate the measured change in scar properties over time. *q* means relative stiffness measure, *B01–B11* indicate patients. ^*^ indicates the regression lines of the corresponding parameters

The ability of the devices to reflect scar maturation in terms of alterations in scar pliability for the POSAS or scar stiffness for the objective suction devices over time was assessed. Figure 4 depicts the relative stiffness measured at the 4 different time points for 9 of the 11 patients in boxplots. For all 3 devices, a tendency towards lower scar stiffness at later time points can be seen. This was specifically assessed using a linear regression analysis. Regression lines were fitted for the longitudinal relative stiffness outcome of each patient. The respective slopes indicated the change over time. In Figure 5, the fits (indicated by asterisks) for each patient with the centered predictor time are shown. We defined the null hypothesis with the slopes avoiding n = 0, indicating no change over the time course. Finally, a one-sided *t*-test with confidence intervals of 95% was used to test for an alternative hypothesis with slope < 0. For these analyses, only 9 out of 11 patients were considered as for 2 patients, only 3 out of 4 measurement visits were completed, leading to their exclusion from this specific assessment. The relative stiffness measures, that is the quotients between scar stiffness and the stiffness of the healthy skin, were used. The results of this analysis are summarized in Table 3.

Compared to the Cutometer, the relative stiffness measured with the Nimble led to a considerably higher intra-time point standard deviation (Figure 4). When analysed with the ICC, an excellent ability to distinguish between individual patients was found for both devices with regard to the healthy skin site: ICC_patient_^Cuto^ = 0.97 and ICC_patient_^Nimble^ = 0.99.

### Comparative analysis of the tools

The Spearman correlation coefficient was used to evaluate the correlation between the stiffness measured by the Nimble, the Cutometer and the POSAS observer and patient pliability scores (Table 4). A moderate correlation was found between the Cutometer and the Nimble and the Cutometer and the POSAS observer pliability score. High correlation was seen between the Nimble and the POSAS observer pliability score and there was weak correlation between the Nimble and the PSOAS patient pliability score. In contrast, no significant correlation could be shown between the patient pliability score and the Cutometer. The patient pliability score demonstrated only weak correlation with the observer pliability score.

**Table 3:** Results of the regression analysis for the ability of the devices to indicate longitudinal change of scar stiffness (for the objective suction devices) or scar pliability (for the POSAS observer pliability score). *p* values and CI as results of the *t*-tests for are shown

**Device**	***P* value**	**CI**
Nimble	0.68E-02	-Inf, −0.128
Cutometer	0.61E-02	-Inf, −0.029
POSAS observer pliability score	0.43E-02	-Inf, −0.115

*POSAS* Patient and Observer Scar Assessment Scale, *CI* confidence interval

**Table 4:** Spearman correlation coefficient between the relative stiffness measures (q) of suction devices and pliability scores of the Patient and Observer Scar Assessment Scale (POSAS). *n* = 42

	***q* ^N^**	***P* value**	***q* ^C^**	***P* value**	**POSAS pliability score**	***P* value**
*q* ^N^	1		0.649	*< 0.001*	0.727	*< 0.001*
*q* ^C^	0.649	*< 0.001*	1		0.579	*< 0.001*
POSAS pliability score	0.727	*< 0.001*	0.579	*< 0.001*	1	
Patient pliability score	0.354	*= 0.02*	0.252	*= 0.11*	0.376	*= 0.01*

### Predictive ability of the suction devices

**Figure 6.**
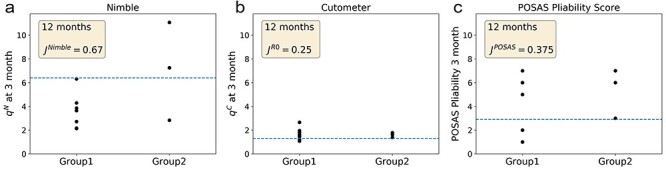
Group classification for prediction of scar outcome at 9 months after injury. **(a)** Skin stiffness ratio *q*^N^ measured by the Nimble at 3 months grouped into Group1 (moderate stiffness scars) and Group2 (high stiffness scars). **(b)** Skin stiffness ratio *q*^C^ measured by the Cutometer and **(c)** Patient and Observer Scar Assessment Scale (POSAS) pliability score results at 3 months grouped into the above-mentioned 2 scar groups. Cut-off values were evaluated with the Youden’s index (J), indicated in each subfigure. *R0* maximum elevation parameter

Next, we determined whether it is possible to predict long-term (12-month) scar stiffness based on an earlier time point (3 months). All scars in our study resulted in similar POSAS pliability scores at 12 months post-injury (Figure 4c). However, when defining a threshold of a POSAS pliability score of 2, 2 groups of scars could be defined. Henceforth, these are referred to as moderate stiffness scars (Group1) and high stiffness scars (Group2). According to this analysis, moderate stiffness scars have a POSAS pliability score ≤2 at 12 months after the injury and high stiffness scars have a POSAS pliability score >2 at the 12-month visit (Figure 6a, b, c). The ROC curve revealed AUC_12m_^N^ = 0.79 for the Nimble, AUC_12m_^POSAS^ = 0.67 for the POSAS pliability score and AUC_12m_^C^ = 0.46 for the Cutometer, indicating a high sensitivity for the Nimble, moderate sensitivity for the POSAS observer pliability score and weak sensitivity for the Cutometer. The most suitable cut-off values for each parameter to differentiate between moderate stiffness and high stiffness scars, calculated by means of the Youden’s index *J*, revealed *J^Nimble^* = 0.67, *J^R0^* = 0.25 and *J^POSAS^* = 0.375, with respective cut-off values of *q^N^* = 6.4, *q^C^* = 1.3 and POSAS pliability = 3.0.

## Discussion

Suction devices have been used to quantify scar pliability, a factor that seemingly determines scar hypertrophy and contraction [47]. Recently, we demonstrated the use of the Nimble, a novel ultra-light suction device, in preclinical and clinical studies on burn patients [35, 37] and patients with systemic sclerosis [38]. The Nimble has been optimized to increase sensitivity and facilitate usability. The present study provides a preliminary assessment of its usefulness for the characterization of scar maturation over time. To this end, we compared 3 quantitative tools to assess their abilities to capture longitudinal changes in biomechanical tissue properties in the first year of scar maturation and predict scar stiffness.

The POSAS, Nimble and Cutometer measurements show distinct longitudinal tendencies (Figure 3); in many patients, scar stiffness increases considerably within the first 6–9 months and it is not until around months 9–12 that a *de novo* recline is observed. In other patients, however, we saw an early and constant decline in stiffness. Exact quantification of these effects remains difficult in our cohort due to the small sample size; however, Figure 3c serves as a rough orientation. Four out of 11 patients showed an increase in the POSAS observer pliability score from month 3 to 6, whereas 5 patients demonstrated a decrease and the remaining 2 an unaltered value. A large study on burn scars by van der Wal *et al.* [17] using similar time points (3, 6 and 12 months) showed an increase in mean POSAS from 3 to 6 months and a decrease afterwards. However, the data provide no indication regarding the course of single patients, impeding direct comparison with our study.

Scar compliance, evaluated by means of the POSAS observer pliability score (Figure 4c) and the Nimble (Figure 4a), improved significantly between 3 and 12 months. The POSAS outcome of our study, with a fairly small sample size, is well in line with the literature. In a study of longitudinal scar assessment [48] in 130 adult burn patients, an improvement of 54% in scar pliability was found with the POSAS (pliability_3m_ = 3.64 (±1.95), pliability_18m_ = 1.95 (±1.07)). Our study revealed a mean improvement of 50%, with pliability_3m_ = 4.63 (±2.1) and pliability_12m_ = 2.27 (±0.75). Previous work also implemented the Cutometer for longitudinal assessment of scars [19, 48]. An improvement of scar compliance parameters for split thickness skin grafts from 3 to 12 months post-injury could be shown, with 22% mean stiffness reduction. In our study the *R0* improved by 33%. The mean *R0* in our study is slightly higher (*R0_3m_* = 1.0 mm (±0.24 mm), *R0_12m_* = 1.33 mm (±0.25 mm)) compared to the values reported previously by others [19, 48]. This difference is most probably due to the different loading protocols, as previous studies commonly used an instant pressure load while in the present study a ramp load was applied, leading to higher strain rates and therefore higher stiffness [49] in the protocol with the instant pressure load. Furthermore, our study accounts for the offset (pre-deformation) in Cutometer measurements by application of the correction procedure as described previously [36].

Spearman correlation coefficients between the POSAS observer pliability score and the scar stiffness measured by suction (Table 4) was moderate, in agreement with Draaijers *et al.* [30] (*r* = 0.53). In contrast, the correlation of the POSAS patient pliability score with the observer pliability score, as well as with the Nimble, was weak and there was no significant correlation between the patient pliability score and the Cutometer. This is not surprising, as previous publications have shown that the patient and the observer perspectives regarding scar severity can differ considerably [50–52] and seem to depend on an array of factors [53, 54]. The present study confirms the correlation of the Nimble and the Cutometer (*r* = 0.649), which is in line with published data on fibrotic skin [37, 38]. In agreement with these previous studies, the Nimble has a high ability to measure differences between the individual patients, with ICC values of ICC_patient_^Nimble^ = 0.99. Finally, we analysed the ability of the applied devices to predict scar quality outcome at 12 months post-injury based on the 3-month visit, resulting in 2 groups (moderate stiffness scars and high stiffness scars). A good ability to predict group assignment at 12 months, based on the 3-month measurement, was observed for the Nimble, moderate ability was found for the standard assessment procedure (the POSAS observer pliability score) and the predictive ability of the Cutometer was found to be weak. The present results are promising and motivate future investigations with larger cohorts. The major limitation of this study is the modest sample size of only 11 pediatric patients. Correction of the data for factors such as performance of skin grafting and age or gender of the patients was not possible, which adds to the high variability of the evaluated time points and could constitute a confounding factor. Subgroup analysis was impossible due to insufficient power. This study mainly demonstrates the applicability of suction-based scar monitoring for a pediatric cohort in a realistic and challenging clinical environment. Another limitation concerns the use of the patient scale of the POSAS, which is intended to be completed by the patient themselves and not a caregiver; however, in several cases in the present study, this was inevitable due to the young ages of the participants.

## Conclusions

Our results indicate that the Nimble and the Cutometer and the POSAS observer pliability score could provide suitable outcome measures for the monitoring of scar maturation in terms of alterations of scar stiffness and pliability, respectively. The Nimble seems particularly well-suited thanks to its shorter measurement duration and ease of use. Both objective suction devices showed excellent abilities to distinguish between individual patients. Furthermore, this preliminary study suggests that all 3 tools might be able to predict scar pliability (POSAS) or stiffness (Cutometer and Nimble) outcome after 12 months post-injury based on an early assessment at 3 months. Further studies with larger sample sizes and a more homogenous cohort are necessary in order to further analyse longitudinal scar formation, verify these preliminary findings and examine the relevance of suction measurements for the clinical assessment of scar maturation.

## Supplementary Material

SupplementaryFig1_tkab028

SupplementaryTable1_tkab028

SupplementaryTable2_tkab028

SupplementaryTable3_tkab028

## Data Availability

Original data not included in the manuscript can be obtained from the authors. Please contact the corresponding author via email.

## Abbreviations

AUC: area under the curve; *h*: predefined elevation; ICC: intraclass correlation coefficient; *J*: Youden’s index; *k*: tissue stiffness; *p_cl_*: closing pressure; *p_max_*: predefined negative pressure; QOL: quality of life; *R0*: maximum elevation parameter; ROC: receiver operating characteristic; *m*: month; *N*: Nimble; *C*: Cutometer.
