# Supplementary figures and images for: Longitudinal monitoring and prediction of long-term outcome of scar stiffness on pediatric patients
Source: Burns Trauma. 2021 Sep 30;9:tkab028. doi: 10.1093/burnst/tkab028 (PMC8484205; doi:10.1093/burnst/tkab028)

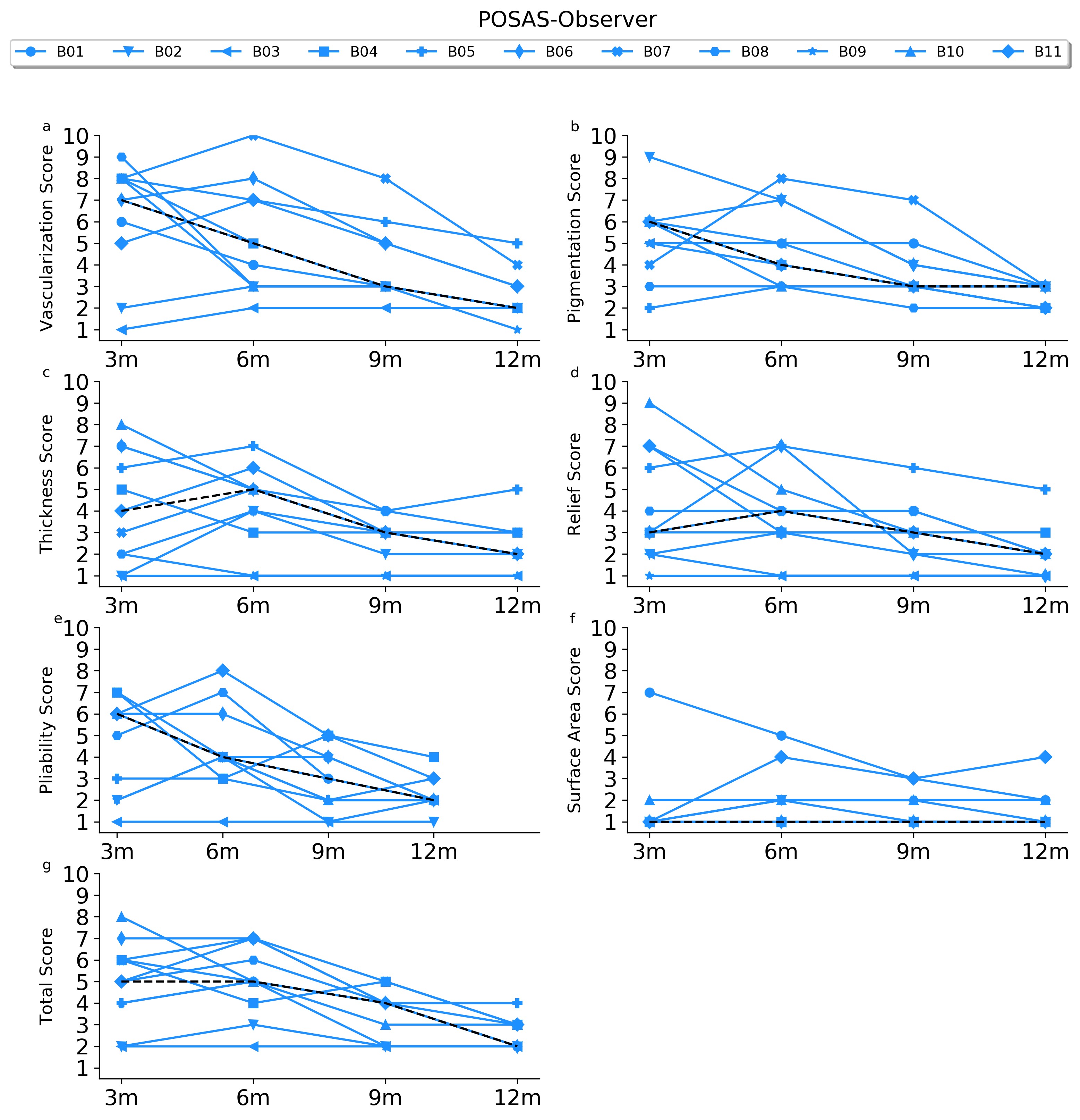

Supplement: SupplementaryFig1_tkab028 [file supplementaryfig1_tkab028.jpeg]
