# Supplementary material for: Longitudinal monitoring and prediction of long-term outcome of scar stiffness on pediatric patients
Source: Burns Trauma. 2021 Sep 30;9:tkab028. doi: 10.1093/burnst/tkab028 (PMC8484205; doi:10.1093/burnst/tkab028)
Supplement: SupplementaryTable1_tkab028 [file supplementarytable1_tkab028.docx]

Supplementary Table 1 Measurement results of the Nimble measurements for each of the 11 patients (B01 – B11) for all the time points (3 months, 6 months, 9 months, and 12 months). The mean and standard deviations of the closing pressures (p_cl_) in mbar and the stiffness parameter (k^Nimble^) in mbar/mm are indicated as well as the data of the quotient – the ratio between the stiffness of the scar tissue to the stiffness of the healthy skin site.

| **Pat.** | **Month** | **Healthy** | | | | **Scar** | | | | **q^N^** |
| --- | --- | --- | --- | --- | --- | --- | --- | --- | --- | --- |
|  |  | **p_cl_ (mbar)** | | **k^Nimble^ (mbar/mm)** | | **p_cl_ (mbar)** | | **k^Nimble^ (mbar/mm)** | | **(-)** |
|  |  | **mean** | **std** | **mean** | **std** | **mean** | **std** | **mean** | **std** |  |
| **B01** | **3** | 10.36 | 1.41 | 20.73 | 2.83 | 37.75 | 15.47 | 75.49 | 30.94 | 3.64 |
|  | **6** | 7.10 | 0.51 | 14.20 | 1.02 | 38.54 | 9.22 | 77.07 | 18.44 | 5.43 |
|  | **9** | 8.30 | 1.06 | 16.61 | 2.12 | 17.09 | 13.04 | 34.17 | 26.09 | 2.06 |
|  | **12** | 16.09 | 0.83 | 32.19 | 1.65 | 21.24 | 6.10 | 42.48 | 12.20 | 1.32 |
| **B02** | **3** | 13.28 | 1.42 | 26.56 | 2.83 | 36.10 | 1.48 | 72.19 | 2.96 | 2.72 |
|  | **6** | 30.47 | 2.27 | 60.94 | 4.54 | 46.19 | 5.36 | 92.37 | 10.72 | 1.52 |
|  | **9** | 29.72 | 2.56 | 59.43 | 5.13 | 19.63 | 5.30 | 39.25 | 10.61 | 0.66 |
|  | **12** | 21.82 | 1.05 | 43.65 | 2.11 | 32.67 | 7.17 | 65.33 | 14.33 | 1.50 |
| **B03** | **3** | 16.27 | 0.97 | 32.55 | 1.95 | 35.21 | 2.56 | 70.41 | 5.11 | 2.16 |
|  | **6** | 27.76 | 4.07 | 55.52 | 8.14 | 31.06 | 10.99 | 62.11 | 21.98 | 1.12 |
|  | **9** | 29.51 | 1.29 | 59.02 | 2.59 | 33.04 | 16.34 | 66.09 | 32.69 | 1.12 |
|  | **12** | 18.08 | 1.14 | 36.17 | 2.28 | 35.58 | 13.03 | 71.17 | 26.06 | 1.97 |
| **B04** | **3** | 10.43 | 0.13 | 20.86 | 0.26 | 75.66 | 26.91 | 151.33 | 53.83 | 7.25 |
|  | **6** | 24.30 | 8.96 | 48.59 | 17.92 | 35.31 | 7.86 | 70.62 | 15.73 | 1.45 |
|  | **9** | 30.88 | 9.38 | 61.77 | 18.75 | 62.14 | 36.85 | 124.29 | 73.70 | 2.01 |
|  | **12** | 18.39 | 1.73 | 36.79 | 3.45 | 35.62 | 2.12 | 71.24 | 4.24 | 1.94 |
| **B05** | **3** | 20.25 | 1.20 | 40.49 | 2.39 | 57.44 | 24.42 | 114.89 | 48.84 | 2.84 |
|  | **6** | 13.21 | 0.56 | 26.43 | 1.12 | 46.22 | 15.17 | 92.45 | 30.35 | 3.50 |
|  | **9** | 18.12 | 2.09 | 36.23 | 4.19 | 16.82 | 7.28 | 33.63 | 14.57 | 0.93 |
|  | **12** | 12.01 | 1.37 | 24.02 | 2.74 | 38.47 | 24.80 | 76.93 | 49.60 | 3.20 |
| **B06** | **3** | 27.11 | 2.29 | 54.22 | 4.58 | 171.23 | 44.00 | 342.47 | 88.00 | 6.32 |
|  | **6** | 31.16 | 10.59 | 62.31 | 21.19 | 137.95 | 38.31 | 275.89 | 76.61 | 4.43 |
|  | **9** | 56.17 | 23.88 | 112.35 | 39.00 | 201.87 | 0.00 | 403.74 | 0.00 | 3.59 |
|  | **12** | 213.92 | 73.72 | 427.83 | 120.39 | nan | nan | nan | nan | nan |
| **B07** | **3** | 29.82 | 1.94 | 59.64 | 3.88 | 127.93 | 51.09 | 255.85 | 102.19 | 4.29 |
|  | **6** | 30.92 | 2.86 | 61.83 | 5.73 | 70.96 | 40.77 | 141.93 | 81.54 | 2.30 |
|  | **9** | 31.31 | 1.46 | 62.63 | 2.92 | 63.07 | 11.98 | 126.14 | 23.95 | 2.01 |
|  | **12** | 27.07 | 1.02 | 54.14 | 2.04 | 23.30 | 7.95 | 46.59 | 15.90 | 0.86 |
| **B08** | **3** | 12.59 | 1.83 | 25.19 | 3.65 | 45.88 | 12.08 | 91.76 | 24.17 | 3.64 |
|  | **6** | 16.09 | 3.83 | 32.19 | 7.67 | 60.29 | 11.77 | 120.58 | 23.54 | 3.75 |
|  | **9** | 10.98 | 1.64 | 21.96 | 3.29 | 24.36 | 5.45 | 48.73 | 10.89 | 2.22 |
|  | **12** | nan | nan | nan | nan | nan | nan | nan | nan | 0.00 |
| **B09** | **3** | 39.84 | 1.09 | 79.68 | 2.18 | 85.20 | 23.70 | 170.41 | 47.40 | 2.14 |
|  | **6** | 38.36 | 1.12 | 76.73 | 2.24 | 72.06 | 13.60 | 144.12 | 27.20 | 1.88 |
|  | **9** | 39.91 | 0.26 | 79.81 | 0.51 | 38.19 | 6.37 | 76.39 | 12.74 | 0.96 |
|  | **12** | 38.23 | 1.27 | 76.45 | 2.53 | 37.99 | 6.33 | 75.97 | 12.66 | 0.99 |
| **B10** | **3** | 20.83 | 4.21 | 41.65 | 8.42 | 80.23 | 30.25 | 160.45 | 60.50 | 3.85 |
|  | **6** | 31.36 | 15.22 | 62.73 | 30.45 | 58.57 | 15.08 | 117.15 | 30.16 | 1.87 |
|  | **9** | 20.56 | 0.63 | 41.11 | 1.26 | 22.96 | 6.33 | 45.91 | 12.67 | 1.12 |
|  | **12** | 72.34 | 6.69 | 144.67 | 13.38 | 112.79 | 65.35 | 225.59 | 130.70 | 1.56 |
| **B11** | **3** | 9.02 | 1.41 | 18.05 | 2.83 | 99.93 | 19.54 | 199.85 | 39.08 | 11.07 |
|  | **6** | 106.82 | 18.38 | 213.64 | 36.76 | 270.85 | 149.19 | 541.69 | 298.37 | 2.54 |
|  | **9** | 21.86 | 1.49 | 43.72 | 2.99 | 36.78 | 15.35 | 73.57 | 30.69 | 1.68 |
|  | **12** | 37.68 | 3.93 | 75.36 | 7.87 | 55.90 | 39.83 | 111.80 | 79.66 | 1.48 |
