# Supplementary material for: Longitudinal monitoring and prediction of long-term outcome of scar stiffness on pediatric patients
Source: Burns Trauma. 2021 Sep 30;9:tkab028. doi: 10.1093/burnst/tkab028 (PMC8484205; doi:10.1093/burnst/tkab028)
Supplement: SupplementaryTable2_tkab028 [file supplementarytable2_tkab028.docx]

Supplementary Table 2 Measurement results of the Cutometer measurements for each of the 11 patients (B01 – B11) for all the time points (3 months, 6 months, 9 months, and 12 months). The mean and standard deviations of the maximum elevation (R0) in mm and the stiffness parameter (k^R0^) in mbar/mm are indicated as well as the data of the quotient – the ratio between the stiffness of the scar tissue to the stiffness of the healthy skin site. Please note that the corrected Cutometer data (R0 and therefore also k^R0^) is shown.

| **Pat.** | **Month** | **Healthy** | | | | **Scar** | | | | **q^C^** |
| --- | --- | --- | --- | --- | --- | --- | --- | --- | --- | --- |
|  |  | **R0 (mm)** | | **k^R0^ (mbar/mm)** | | **R0 (mm)** | | **k^R0^ (mbar/mm)** | | **(-)** |
|  |  | **mean** | **std** | **mean** | **std** | **mean** | **std** | **mean** | **std** |  |
| B01 | 3 | 1.879 | 0.196 | 134.420 | 13.359 | 1.030 | 0.050 | 243.244 | 12.336 | 1.810 |
|  | 6 | 1.972 | 0.031 | 126.807 | 2.026 | 1.318 | 0.106 | 190.807 | 14.614 | 1.505 |
|  | 9 | 1.875 | 0.076 | 133.562 | 5.603 | 1.590 | 0.172 | 159.036 | 16.421 | 1.191 |
|  | 12 | 2.053 | 0.178 | 122.664 | 10.070 | 1.681 | 0.102 | 149.290 | 9.364 | 1.217 |
| B02 | 3 | 1.549 | 0.156 | 163.021 | 16.586 | 1.444 | 0.094 | 173.859 | 11.809 | 1.066 |
|  | 6 | 1.601 | 0.308 | 161.949 | 30.543 | 1.193 | 0.196 | 215.976 | 39.543 | 1.334 |
|  | 9 | 1.185 | 0.032 | 211.065 | 5.675 | 1.381 | 0.065 | 181.460 | 8.287 | 0.860 |
|  | 12 | 1.508 | 0.089 | 166.419 | 10.148 | 1.142 | 0.161 | 223.871 | 35.110 | 1.345 |
| B03 | 3 | 1.359 | 0.049 | 184.203 | 6.790 | 1.151 | 0.212 | 223.997 | 37.036 | 1.216 |
|  | 6 | 1.518 | 0.001 | 164.654 | 0.135 | 1.387 | 0.078 | 180.871 | 10.397 | 1.098 |
|  | 9 | 1.286 | 0.034 | 194.492 | 5.286 | 1.254 | 0.072 | 200.092 | 11.714 | 1.029 |
|  | 12 | 1.633 | 0.145 | 154.365 | 14.653 | 1.398 | 0.041 | 178.982 | 5.319 | 1.159 |
| B04 | 3 | 1.408 | 0.085 | 178.195 | 11.251 | 1.043 | 0.165 | 246.527 | 43.734 | 1.383 |
|  | 6 | 1.389 | 0.039 | 180.168 | 4.984 | 0.925 | 0.179 | 282.354 | 63.065 | 1.567 |
|  | 9 | 1.194 | 0.006 | 209.385 | 1.005 | 0.964 | 0.132 | 263.772 | 33.019 | 1.260 |
|  | 12 | 1.459 | 0.115 | 172.388 | 13.180 | 1.062 | 0.071 | 236.523 | 15.686 | 1.372 |
| B05 | 3 | 2.143 | 0.177 | 117.525 | 10.252 | 1.337 | 0.144 | 189.363 | 21.654 | 1.611 |
|  | 6 | 2.125 | 0.100 | 117.906 | 5.491 | 1.032 | 0.290 | 260.849 | 67.432 | 2.212 |
|  | 9 | 2.272 | 0.138 | 110.473 | 6.942 | 1.556 | 0.049 | 160.830 | 5.129 | 1.456 |
|  | 12 | 2.303 | 0.410 | 112.556 | 22.594 | 1.787 | 0.586 | 156.242 | 51.246 | 1.388 |
| B06 | 3 | 1.818 | 0.110 | 138.020 | 8.340 | 0.714 | 0.138 | 365.948 | 81.849 | 2.651 |
|  | 6 | 1.137 | 0.076 | 220.828 | 14.161 | 0.634 | 0.098 | 405.346 | 70.005 | 1.836 |
|  | 9 | 1.276 | 0.155 | 198.066 | 21.096 | 0.665 | 0.064 | 378.056 | 29.203 | 1.909 |
|  | 12 | 1.267 | 0.063 | 178.599 | 32.445 | 0.738 | 0.020 | 338.919 | 7.502 | 1.898 |
| B07 | 3 | 1.275 | 0.044 | 196.308 | 6.639 | 0.689 | 0.181 | 385.010 | 85.183 | 1.961 |
|  | 6 | 1.673 | 0.085 | 149.807 | 7.363 | 0.923 | 0.174 | 279.744 | 47.924 | 1.867 |
|  | 9 | 1.766 | 0.061 | 141.704 | 4.817 | 1.066 | 0.163 | 239.503 | 33.114 | 1.690 |
|  | 12 | 1.482 | 0.163 | 170.669 | 18.382 | 1.304 | 0.174 | 195.270 | 26.678 | 1.144 |
| B08 | 3 | 1.729 | 0.134 | 145.466 | 11.274 | 1.138 | 0.168 | 224.578 | 33.154 | 1.544 |
|  | 6 | 1.856 | 0.171 | 135.947 | 13.271 | 1.057 | 0.090 | 238.123 | 19.687 | 1.752 |
|  | 9 | 1.913 | 0.046 | 130.761 | 3.182 | 1.170 | 0.043 | 213.967 | 7.965 | 1.636 |
|  | 12 | nan | nan | nan | nan | nan | nan | nan | nan | nan |
| B09 | 3 | 1.216 | 0.034 | 205.751 | 5.759 | 0.842 | 0.058 | 298.429 | 20.271 | 1.450 |
|  | 6 | 1.479 | 0.053 | 169.292 | 6.130 | 0.973 | 0.087 | 259.039 | 24.591 | 1.530 |
|  | 9 | 1.486 | 0.008 | 168.280 | 0.926 | 1.242 | 0.085 | 202.336 | 14.527 | 1.202 |
|  | 12 | 1.526 | 0.081 | 164.292 | 8.721 | 1.253 | 0.101 | 200.766 | 15.461 | 1.222 |
| B10 | 3 | 1.170 | 0.098 | 215.116 | 17.172 | 0.722 | 0.115 | 354.606 | 53.423 | 1.648 |
|  | 6 | 1.212 | 0.099 | 207.767 | 17.713 | 0.923 | 0.030 | 271.299 | 8.970 | 1.306 |
|  | 9 | 1.261 | 0.053 | 198.549 | 8.269 | 0.763 | 0.040 | 328.714 | 17.620 | 1.656 |
|  | 12 | 1.357 | 0.061 | 184.615 | 8.565 | 1.000 | 0.124 | 254.220 | 33.538 | 1.377 |
| B11 | 3 | 1.645 | 0.175 | 153.694 | 15.852 | 0.925 | 0.092 | 272.993 | 28.543 | 1.776 |
|  | 6 | 1.257 | 0.176 | 202.526 | 26.369 | 0.963 | 0.266 | 286.645 | 97.633 | 1.415 |
|  | 9 | 1.362 | 0.113 | 184.710 | 14.485 | 1.344 | 0.139 | 187.937 | 18.583 | 1.017 |
|  | 12 | 1.351 | 0.147 | 187.337 | 21.130 | 1.369 | 0.219 | 187.193 | 29.066 | 0.999 |
