# Supplementary material for: Longitudinal monitoring and prediction of long-term outcome of scar stiffness on pediatric patients
Source: Burns Trauma. 2021 Sep 30;9:tkab028. doi: 10.1093/burnst/tkab028 (PMC8484205; doi:10.1093/burnst/tkab028)
Supplement: SupplementaryTable3_tkab028 [file supplementarytable3_tkab028.docx]

Supplementary Table 3 Results of the POSAS assessment for each of the 11 patients (B01 – B11) for all the time points (3 months, 6 months, 9 months, and 12 months). The score value of the individual scar properties (vascularity, pigmentation, thickness, relief, surface area, pliability and total score) are indicated.

| **Pat.** | **Month** | **Vascularity** | **Pigmentation** | **Thickness** | **Relief** | **Surface area** | **Pliability** | **Total** |
| --- | --- | --- | --- | --- | --- | --- | --- | --- |
| **B01** | **3** | 6 | 6 | 7 | 7 | 7 | 6 | 6 |
|  | **6** | 4 | 5 | 5 | 4 | 5 | 4 | 5 |
|  | **9** | 3 | 5 | 3 | 4 | 3 | 3 | 4 |
|  | **12** | 2 | 3 | 3 | 2 | 2 | 2 | 2 |
| **B02** | **3** | 2 | 9 | 1 | 2 | 1 | 2 | 2 |
|  | **6** | 3 | 7 | 4 | 3 | 2 | 4 | 3 |
|  | **9** | 3 | 4 | 2 | 2 | 1 | 1 | 2 |
|  | **12** | 2 | 3 | 2 | 2 | 1 | 1 | 2 |
| **B03** | **3** | 1 | 5 | 1 | 2 | 1 | 1 | 2 |
|  | **6** | 2 | 5 | 1 | 1 | 1 | 1 | 2 |
|  | **9** | 2 | 3 | 1 | 1 | 1 | 1 | 2 |
|  | **12** | 2 | 3 | 1 | 1 | 1 | 2 | 2 |
| **B04** | **3** | 8 | 6 | 5 | 3 | 1 | 7 | 6 |
|  | **6** | 5 | 4 | 3 | 3 | 1 | 3 | 4 |
|  | **9** | 3 | 3 | 3 | 3 | 1 | 5 | 5 |
|  | **12** | 2 | 2 | 3 | 3 | 1 | 4 | 3 |
| **B05** | **3** | 8 | 2 | 6 | 6 | 1 | 3 | 4 |
|  | **6** | 7 | 3 | 7 | 7 | 1 | 3 | 5 |
|  | **9** | 6 | 3 | 4 | 6 | 1 | 2 | 4 |
|  | **12** | 5 | 3 | 5 | 5 | 1 | 3 | 4 |
| **B06** | **3** | 7 | 6 | 7 | 3 | 1 | 6 | 7 |
|  | **6** | 8 | 7 | 5 | 7 | 1 | 6 | 7 |
|  | **9** | 5 | 4 | 3 | 2 | 1 | 4 | 4 |
|  | **12** | 3 | 3 | 2 | 1 | 1 | 2 | 2 |
| **B07** | **3** | 8 | 4 | 3 | 3 | 1 | 7 | 6 |
|  | **6** | 10 | 8 | 5 | 4 | 1 | 4 | 7 |
|  | **9** | 8 | 7 | 4 | 4 | 1 | 4 | 5 |
|  | **12** | 4 | 3 | 3 | 2 | 1 | 2 | 3 |
| **B08** | **3** | 9 | 3 | 2 | 4 | 1 | 5 | 5 |
|  | **6** | 3 | 3 | 4 | 4 | 2 | 7 | 6 |
|  | **9** | 3 | 2 | 3 | 3 | 2 | 3 | 4 |
|  | **12** | 2 | 2 | 2 | 2 | 1 | 2 | 2 |
| **B09** | **3** | 7 | 5 | 2 | 1 | 1 | 2 | 4 |
|  | **6** | 5 | 4 | 1 | 1 | 1 | 4 | 5 |
|  | **9** | 3 | 3 | 1 | 1 | 1 | 2 | 2 |
|  | **12** | 1 | 2 | 1 | 1 | 1 | 2 | 2 |
| **B10** | **3** | 8 | 6 | 8 | 9 | 2 | 6 | 8 |
|  | **6** | 3 | 3 | 5 | 5 | 2 | 4 | 5 |
|  | **9** | 3 | 3 | 3 | 3 | 2 | 2 | 3 |
|  | **12** | 2 | 3 | 2 | 2 | 2 | 2 | 3 |
| **B11** | **3** | 5 | 6 | 4 | 7 | 1 | 6 | 5 |
|  | **6** | 7 | 4 | 6 | 3 | 4 | 8 | 7 |
|  | **9** | 5 | 3 | 3 | 3 | 3 | 5 | 4 |
|  | **12** | 3 | 2 | 2 | 2 | 4 | 3 | 3 |
